# Simultaneous Stenting With Edwards SAPIEN Transcatheter Pulmonary Valve Replacement

**DOI:** 10.1016/j.jscai.2022.100553

**Published:** 2022-12-08

**Authors:** Jessica Heibel, Ryan Reeves, Laith Alshawabkeh, Henri Justino, Howaida El-Said

**Affiliations:** a Cardiology, Rady Children’s Hospital, University of California San Diego, San Diego, California; b Division of Cardiovascular Medicine, University of California San Diego, San Diego, California

**Keywords:** SAPIEN valve, simultaneous stenting, transcatheter pulmonary valve replacement

## Abstract

**Background:**

Prestenting of the landing zone for transcatheter pulmonary valve replacement (TPVR) with a balloon-expandable valve can dilate a stenotic right ventricular outflow tract (RVOT), prevent paravalvar leak (PVL), and protect against conduit tear. Simultaneous stenting (SS) with the Melody valve has been described, but to our knowledge, SS with a SAPIEN valve has not been reported. We report our experience with this novel technique.

**Methods:**

A retrospective chart review of patients who underwent TPVR at Rady Children’s hospital and UCSD Medical Center was performed. Patients were included if they had underwent SAPIEN TPVR with SS. Rationale for stent choice was a bare metal stent to relieve long-segment stenosis and covered stents to prevent PVL or to protect against conduit tear.

**Results:**

A total of 17 cases were identified. The majority of RVOTs were transannular patches (n = 9, 56%), with a minimum diameter of 19.6 ± 5.2 mm, and the most common valve placed was an Edwards SAPIEN 26.0 mm (n = 10, 59%). All SAPIEN valves placed were of the S3 generation. The procedure was successful in all patients, with no conduit tears. Minor complications occurred in 3 patients (17.6%).

**Conclusions:**

Simultaneous stent deployment with a SAPIEN TPVR is an alternative 1-step technique for patients who require prestenting. SS simplifies the procedure, has low complication rates, and offers the benefits of a longer landing zone and decreased PVL.

## Highlights

• Simultaneous stenting with the SAPIEN valve is technically feasible and safe.
• Simultaneous stenting offers a longer landing zone in long-segment stenosis.
• Protects against dissection and paravalvar leak.
• Up to 3 additional stents can be deployed simultaneously within a 26F DrySeal sheath catheter.

## Introduction

Nonsurgical treatment of dysfunctional right ventricular outflow tracts (RVOTs), right ventricular-to-pulmonary artery conduits, and bioprosthetic valves has been safely accomplished for over 20 years with transcatheter pulmonary valve replacement (TPVR).^1^, ^2^, ^3^, ^4^, ^5^, ^6^ Different valve options available include balloon-expandable valves (Melody [Medtronic] and Edwards SAPIEN transcatheter heart valve [Edwards Lifesciences]) and self-expanding platforms for large diameter RVOTs (Medtronic Harmony [Medtronic], Alterra Adaptive Prestent [Edwards Lifesciences], and Venus P-valve [Venus Medtech]).

Stent placement before valve insertion, or prestenting, became the standard of care with the introduction of Melody valve to prevent fracturing of the Melody stent.^7^ Similarly, prestenting with the SAPIEN valve is used to prepare stenotic RVOTs or conduits and to prevent against conduit tear and/or paravalvar leak (PVL).

Previously, a “1-step” technique has been reported in which stents were simultaneously deployed with the Melody valve on the Ensemble delivery system (Medtronic).^8^ Outcomes using this technique were comparable with those of other studies, with significant reduction in procedural time and radiation dose. Our center expanded on this technique with various combinations of bare metal and covered stents mounted on the Melody valve and deployed simultaneously.^9^

Continuing with this experience, we applied the 1-step technique to TPVR with SAPIEN valves. Here, we report our single-center experience with simultaneous stenting (SS) and SAPIEN valve deployment.

## Methods

Patients who underwent SAPIEN TPVR with SS from 2018 to 2021 at Rady Children’s Hospital and University of California San Diego were included in this retrospective analysis. Institutional review board approval from University of California San Diego was obtained.

### Procedure description

**Figure 1:**
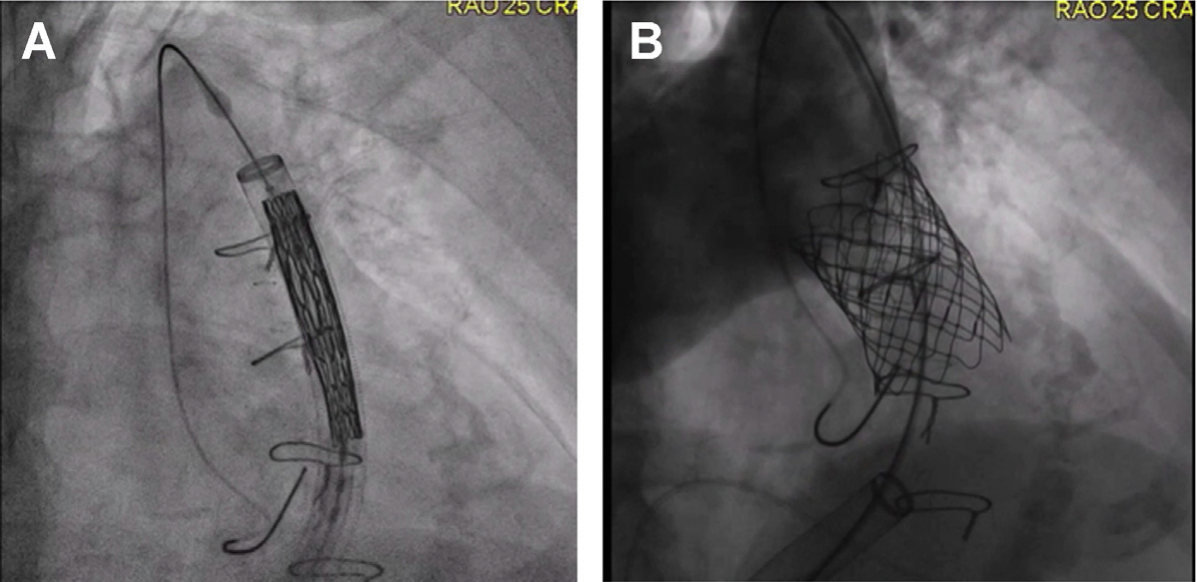
**SAPIEN valve and SS stent deployment through DrySeal sheath.** (**A**) Predeployment within the right ventricular outflow tract; (**B**) postdeployment. SS, simultaneous stenting.

Initial steps for SS in SAPIEN valve implantation are similar to those of conventional SAPIEN valve implantation. Before intervention, coronary arteries are evaluated with 3-dimensional rotational angiography and computed tomography and balloon sizing with simultaneous coronary angiography or a combination of these methods. Balloon sizing is important with this method when choosing valve size, location, and type/number of stents. A compliant sizing balloon is inflated in the conduit/RVOT to evaluate the length of stenosis, minimal diameter, and relation of the targeted landing zone to the coronary arteries. However, sequential inflation and compliance testing is not typically performed in our center. The valve is then prepared as usual on the delivery system using the Edwards crimper. Then, the chosen stent is partially dilated to fit easily over the mounted valve and crimped down using the Edwards crimping device. A Coombs dilator (Cook Medical) or 3.0 to 4.0-mm balloon can be used to dilate the stent, so that it is large enough to slip over the mounted valve without disrupting it or damaging the stent. This step is repeated for additional stents, as necessary (Central Illustration). The stents are usually offset compared with the valve, so the valve is deployed within the proximal portion of the stent(s). Offsetting in this fashion prevents additional stent length from overhanging into the RVOT, reducing risk of fracture and ectopy. If there is a long-segment stenosis present that surpasses the length of available stents, then overlapping stents have been used to expand this length. The area of interest is crossed using a 26F, 60.0-cm DrySeal sheath (W.L Gore). Initial advancement of the DrySeal past the interventional area is a key step. If the sheath cannot advance through the area, then the delivery ensemble will not be able to either. The entire delivery system with valve and stents is inserted into the 26F sheath and deployed under fluoroscopic and angiographic guidance (Figure 1), with postdilation if needed. In the cases in which the lengths of additional stents exceeded the lengths of the delivery balloon, the balloon is rapidly deflated, adjusted to nonexpanded stents, and reinflated. The delivery balloon has been adequate to deploy the valve with additional stents without adjustment to recommended inflation volumes. However, occasional postdilation with an additional noncompliant balloon has been performed in a few cases.

**Central Illustration:**
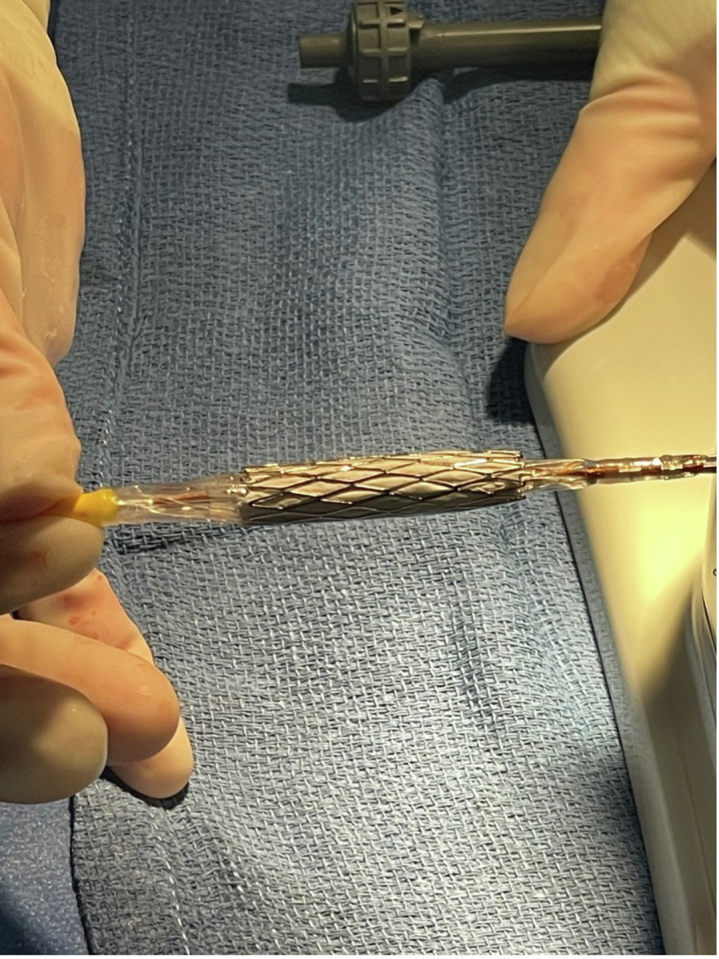
A covered stent, followed by a bare metal stent, are sequentially mounted on an Edwards SAPIEN valve in preparation for simultaneous stenting in transcatheter pulmonary valve replacement.

### Statistical analysis

Patients were included in the analysis if they had underwent TPVR with a SAPIEN valve and simultaneous stent deployment. Patient characteristics and procedural outcomes were compiled, with categorical variables reported as frequencies and percentages and continuous variables as means and standard deviations. Owing to a small sample size, the Fisher exact test was used to compare categorical variables from larger studies.

## Results

Patient demographics and outcomes are reported in Table 1. A total of 17 cases were identified in 16 patients (1 returned for a second valve placement). The mean age was 25 years (SD 9 years; range 9-38 years), and 11 (65%) were male. Original cardiac diagnoses were tetralogy of Fallot (n = 7, 44%), pulmonary atresia with intact ventricular septum (n = 3, 19%), double outlet right ventricle (n = 3, 19%), bicuspid aortic valve (n = 2, 13%), and truncus arteriosus (n = 1, 6%). Most RVOTs were transannular patches (n = 9, 56%), followed by pulmonary homograft (n = 4, 25%), and Contegra conduits (n = 3, 19%), with a minimum preimplant diameter of 19.6 ± 5.2 mm. Most of the TPVR referrals were for pulmonary insufficiency (n = 8, 47%), followed by mixed insufficiency and stenosis (n = 6, 35%) and pulmonary stenosis (n = 3, 18%), and almost half of the patients reported initial right ventricular pressure levels more than one-half of systemic pressure levels (n = 7, 41%). Valve size chosen for implant ranged from 23.0 mm to 29.0 mm, and the most commonly valve placed was a 26.0-mm Edwards SAPIEN S3 (n = 10, 59%). Stents deployed were bare metal (n = 6, 35%), covered Cheatham platinum (CP) (n = 6, 35%), and a combination of bare metal and covered CP stents (n = 5, 30%). The procedure was successful in all patients, with no conduit tears and all gradient pressure levels <10 mm Hg. Complications occurred in 3 patients (17.6%, 2 major and 1 minor), which included 1 (6%) valve migration, 1 (6%) PVL, and 1 (6%) postoperative valvar stenosis. Valve migration occurred because of undersizing the valve to avoid compression of the coronary artery. The valve was captured more proximally within the RVOT, and subsequent valve deployment was larger because the coronary artery was further removed at the new landing zone. The PVL occurred owing to a tear in the covering of the CP stent, necessitating additional stent and valve deployment. The stent fracture occurred postoperatively because to a covered CP stent extending into the RVOT, which caused subvalvar stenosis, requiring repeat TPVR 7 months later. The repeat procedure is included in this cohort (to explain: patients, n = 16; cases, n = 17). All these complications occurred earlier in our experience. The Fisher exact test showed no significant difference (*P* = .18) when comparing complication rates with those of a larger muticenter study.^10^

**Table 1:** Patient demographics, characteristics, procedural details, and outcomes from simultaneous stenting with Edwards SAPIEN valve

Patients	n = 16
Male sex	11 (65%)
Age, y	25 ± 9
Diagnosis	n = 16
Tetralogy of Fallot	7 (44%)
Pulmonary atresia/intact ventricular septum	3 (19%)
Double outlet right ventricle	3 (19%)
Bicuspid aortic valve	2 (12%)
Truncus arteriosus	1 (6%)
Type of RVOT	n = 16
Native, transannular patch	9 (56%)
Pulmonary homograft	4 (25%)
Contegra conduit	3 (19%)
Indication	n = 17
PI	8 (47%)
PS	3 (18%)
PI and PS	6 (35%)
Calcification of conduit/RVOT	n = 16
Severe	4 (25%)
Moderate	3 (19%)
Mild	1 (6%)
None	8 (50%)
Preoperative hemodynamics	
RVP ≥1/2 SBP	7 (41%)
Minimum RVOT size, mm	19.6 ± 5.2
Edwards valve size, mm	
23	3 (17.5%)
26	10 (59%)
29	4 (23.5%)
Stent type	
Bare metal	6 (35%)
Covered CP stent	6 (35%)
Bare metal + covered CP stent	5 (30%)
Postoperative hemodynamics	
PI ≥ mild	0 (0%)
PV gradient <10 mm Hg	17 (100%)
Complications	n = 3 (17.6%)
Valve migration	1
Valve stenosis	1
Paravalvar leak	1

CP, Cheatham platinum; PI, pulmonary insufficiency; PS, pulmonary stenosis; PV, pulmonary valve; RVOT, right ventricular outflow tract; RVP, right ventricular pressure; SBP, systolic blood pressure.

## Discussion

Simultaneous stenting is a safe, technically feasible method for prestenting in TPVR with SAPIEN valves. This case series builds on our center’s prior experience of prestenting with the Melody valve, which has previously demonstrated reduced radiation dose and contrast use. Similar benefits were expected for this cohort, although not directly evaluated in this study. In addition, adopting the 1-step technique can be useful when crossing the RVOT is difficult or there might be concern for dislodging the prestent.

Our rate of complications was comparable with that of a large, multicenter study.^10^ We anticipate further reduction in complication rates as we have modified our approach with experience as described further.

**Figure 2:**
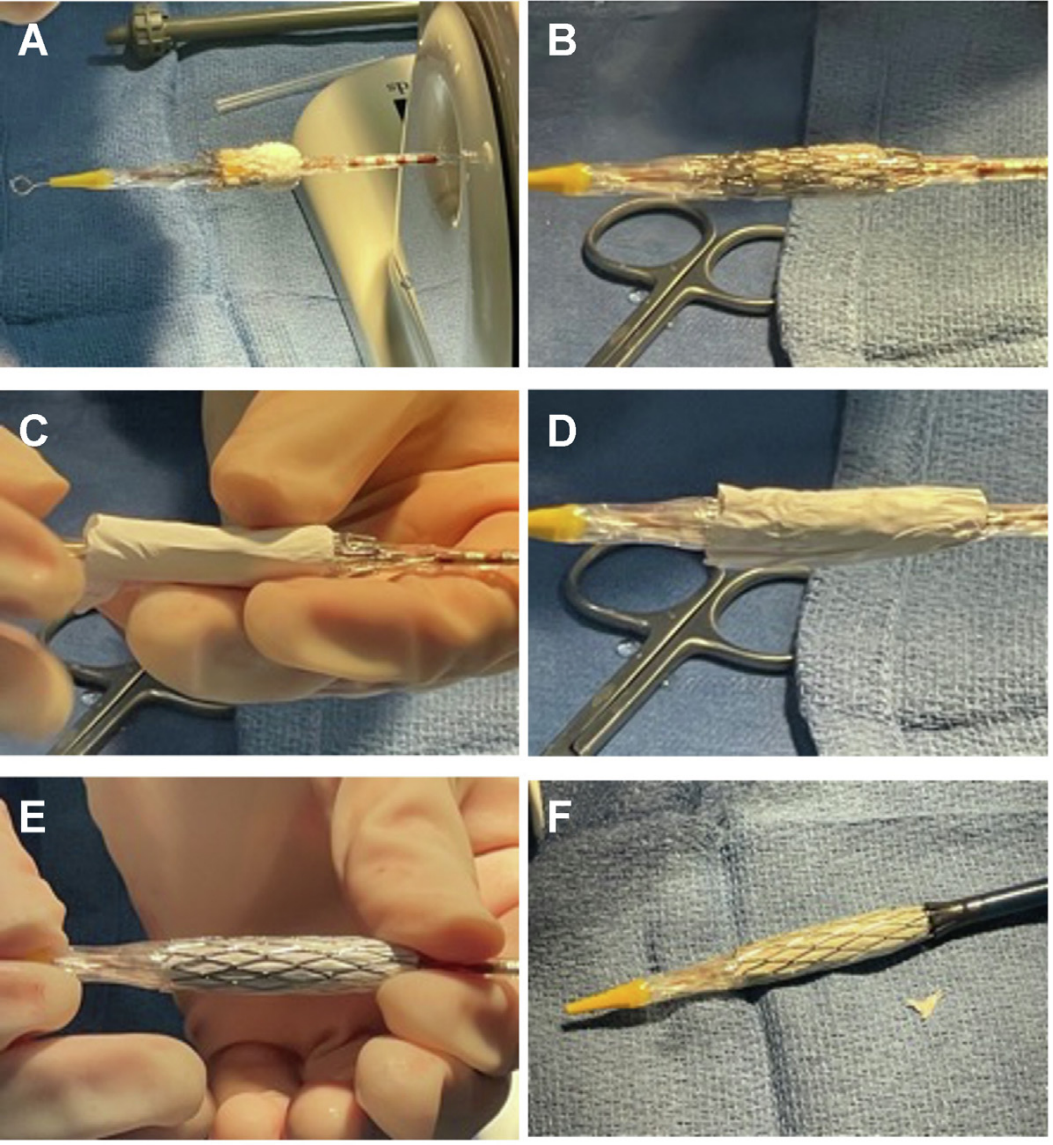
**Description of valve and stent mounting technique for simultaneous deployment.** The Edwards SAPIEN valve is first mounted on the delivery system (**A**), followed by a bare metal (**B**), the covered stent (**C, D**), and, finally, an additional bare metal (**E**) to stabilize cover during insertion. (**F**) Final appearance before insertion of the delivery system through 26F DrySeal sheath with the valve/stent complex crimped directly on the Edwards deployment balloon.

We have previously reported our reasoning behind stent selection^9^ with some modifications to SAPIEN SS deployment. In brief, for conduits, covered CP stents are chosen to protect against conduit tear, especially in severely stenotic or calcified conduits. If the length of stenosis exceeds that of the valve frame, then a bare metal stent is added to span the stenotic area and for increased radial strength to prevent recoil. In modified and patched native RVOTs, SS was used in patients with short landing zones when solo deployment of the SAPIEN valve would otherwise be difficult. SS with a covered CP stent extends the landing zone to protect against PVL. The increased radial strength of the Palmaz XD stent protects against CP stent fracture and subvalvar stenosis. Owing to propensity of CP stent subvalvar fracture–causing stenosis, we recommend always pairing a CP stent with a bare metal stent if part of the CP stent will extend past the SAPIEN valve into the RVOT. The case with PVL occurred despite the use of a covered CP stent. The stent was adequately placed and should have prevented a leak; however, it was an 8-zig stent with less polytetrafluoroethylene material. We suspect the covering tore as the 8 zigs were overexpanded. Currently, we use only the 10-zig covered CP stents, which has a longer covering to accommodate the larger maximum expanded diameter. To further prevent against tearing or displacement of the polytetrafluoroethylene during deployment, when opting to use 2 bare metal stents, we sandwich the CP covered stent between the 2 Palmaz XD stents (Figure 2). No further PVLs or valvar stenosis have occurred after these modifications, which were implemented early in our experience.

With the strong radial force supplied by the cobalt-chromium stainless steel frame of the SAPIEN valve family, prestenting for TPVR is not always indicated.^11^ Compared with that of the Melody valve, the SAPIEN fracturing and recoiling of the frame is greatly reduced although not eliminated. Prestenting is still useful in various scenarios, as shown in the study by Morgan et al,^11^ in which 10 of the 71 cases underwent prestenting. With long-segment RVOT or conduit stenosis, because the valve frame height ranges from 14.0 to 22.5 mm for the 23.0-mm to 29.0-mm valves, respectively,^12^ it is necessary to prestent if the stenotic area exceeds these valve heights. In this case series, the Palmaz XD stents was used to span long-segment stenoses. Otherwise, obstruction was not relieved, and there was a suboptimal TPVR result. In addition, in heavily calcified conduits/homografts, we anticipate the need for greater radial strength and use additional Palmaz XD stents to compensate for this. Similar considerations need to be considered to avoid PVL. The height of the 29-mm SAPIEN valve covering is 11.6 mm for both the XT and S3 generations, with shorter coverings for the smaller valves. If the targeted landing zone is similar or less than this length, then prestenting with a covered stent eases valve delivery and protects against paravalvar leak by extending the landing zone. Moreover, covered stents are recommended for heavily calcified conduits for which dissection is a concern.^13^

A limitation of this technique is the reliance on operator expertise and predicting which stents are needed for prestenting. If the chosen stents do not relieve stenosis owing to recoiling or there is a conduit tear that is not protected by a covered stent, then the valve is wasted because additional stents would then be deployed within it. Implanting a second valve for successful completion of TPVR significantly increases the cost of the procedure. Because the 26F delivery sheath accommodates additional stents, we recommend being aggressive in stent usage and adding an additional stent if there is concern for noncompliant conduit or potential tear.

### Study limitations

This study has the inherent limitations of a retrospective chart review and small cohort. We did not include a comparison cohort without SS or with other valves used in TPVR. To compensate, we compared against a multiinstitutional cohort to verify outcomes and complication rates that were similar to this standard.

## Conclusions

Simultaneous stenting for TPVR with SAPIEN valves is technically feasible with up to 3 stents and various combinations. We did not find a significant difference in complications compared with those of a larger cohort and, hence, extrapolate that this 1-step technique reduces radiation dose and contrast use as shown in similar previous studies. Further studies would benefit from a larger population, with a matched cohort for non-SS and for other valves used in TPVR, to more clearly demonstrate whether differences in outcomes or safety are noted with this technique.
